# Trends in the prevalence of cardiovascular disease, defined as ECG abnormalities and/or self-reported events, in Mauritius between 1987 and 2021: analysis of data from seven large population-based surveys

**DOI:** 10.1136/bmjopen-2024-087693

**Published:** 2025-06-16

**Authors:** Stefan Söderberg, Hina Taki, Sudhir Kowlessur, Jonathan E Shaw, Dianna J Magliano, Abdonas Tamosiunas, George Alberti, Paul Zimmet, Jaakko Tuomilehto

**Affiliations:** 1 Department of Public Health and Clinical Medicine, Umeå University, Umea, Sweden; 2 NCD Unit, Minsitry of health and Wellness, Port Louis, Mauritius; 3 Baker Heart and Diabetes Institute, Melbourne, Victoria, Australia; 4 Lithuanian University of Health Sciences, Kaunas, Lithuania; 5 Department of Endocrinology and Metabolism, Imperial College London, London, UK; 6 Department of Diabetes, Monash University, Melbourne, Victoria, Australia; 7 Department of Public Health, University of Helsinki, Helsinki, Finland

**Keywords:** Cardiac Epidemiology, Myocardial infarction, Coronary heart disease

## Abstract

**Abstract:**

**Objective:**

To estimate the prevalence of coronary heart disease (CHD) in Mauritius. Over the last half century, rapid socioeconomic development has taken place in the multiethnic Mauritius. It is unclear if this is paralleled with an increasing prevalence of CHD.

**Design:**

Repeated cross-sectional population-based studies.

**Setting:**

Mauritius.

**Participants:**

Seven population-based surveys were performed in Mauritius between 1987 and 2021. Altogether, 29 997 participants aged 35–74 years were included.

**Measures:**

Except in 2004 and 2021, all participants were examined with an ECG. ECG changes were classified as ‘probable CHD’ and ‘possible CHD’ according to the Minnesota Code model. Participants were asked about previous myocardial infarction, stroke and angina pectoris as told by a doctor. An affirmative answer to any of these questions was labelled as the presence of cardiovascular disease (CVD). Since 2009, questions about previous coronary bypass surgery and percutaneous coronary intervention were included. The prevalence estimates were age and sex standardised to the 2008 Mauritian population. Multivariable logistic regression evaluated associations between traditional CVD risk factors and CHD.

**Results:**

The prevalence (with 95% CI) of probable CHD according to ECG did not increase between 1987 and 2015, 1.6% (1.2–2.1%) and 1.9% (1.5–2.3%), respectively, whereas the prevalence of possible CHD decreased, 23.7% (22.3–25.1%) and 17.3% (16.2–18.3%), respectively. Self-reported CVD did not increase between 1987 and 2021. Male sex, diabetes, impaired glucose tolerance (IGT), hypertension, smoking and self-reported history of CVD were associated independently with probable CHD, whereas female sex, IGT, hypertension, high cholesterol and self-reported history of CVD were associated independently with possible CHD. Ethnicity did not associate with probable CHD but with possible CHD. Postload plasma glucose associated with probable and possible CHD.

**Conclusions:**

The prevalence of probable CHD according to ECG and the prevalence of self-reported history of CVD did not increase in Mauritius. Traditional cardiovascular risk factors were associated significantly with the presence of probable and possible CHD.

**STRENGTHS AND LIMITATIONS OF THIS STUDY**

- The Mauritius Non-Communicable Disease (NCD) study has surveyed a population with three ethnicities representing large population groups globally.
- The Mauritius NCD study is population-based and covers a long period of time.
- The methodology has been consistent since the start (ECG coding, oral glucose tolerance test, definitions).
- The major limitations are selection and survival bias, as those with manifest cardiovascular disease may not survive until the next health survey, or they may not attend due to already established contact with healthcare.
- ECG abnormalities were used as a proxy for coronary heart disease (CHD), but the true presence of CHD was not verified.

## Introduction

 Cardiovascular disease (CVD) is the most common cause of death globally. By 2030, 80% of all CVD deaths will occur in low- and middle-income countries (LMICs).^1^,^3^ However, there are limited data on the prevalence and trends of CVD based on reliable methods.

There are many reasons for this anticipated scenario, including the increase in life expectancy providing longer periods of exposure to CVD risk factors, mainly due to the decline in deaths occurring in infancy and youth.^4^ Another reason is changes in lifestyle resulting from globalisation and urbanisation leading to increased intake of energy-dense foods, decrease in physical activity and increased sedentary behaviour and psychosocial stress. These changes promote higher risk factor levels, such as smoking, obesity, type 2 diabetes, dyslipidaemia and hypertension.^5^

CVD is a major disease in the epidemiological transition to non-communicable diseases (NCDs),^6^ and CVD has become the leading cause of death in LMICs.^4^ It is a dynamic process where different countries are progressing at different paces. In LMCIs where routine health register data of coronary heart disease (CHD) are scarce, cross-sectional studies of ECG abnormalities may provide a valuable way to estimate the occurrence of CHD.

The island nation of Mauritius has gone through a profound transition with changing living conditions and lifestyles accompanied by changes in disease burden due to chronic NCDs such as CVD and diabetes.^7^ Mauritius has followed this process through repeated cross-sectional surveys since the mid-‘80s.^8^ In this report, we describe trends in the prevalence of self-reported CVD and ECG abnormalities indicative of CHD in Mauritius. Our a priori hypothesis was that the prevalence of CVD may increase in parallel with increasing prevalence of traditional cardiovascular risk factors.

## Methods

### Population and survey methodology

Mauritius is located in the southern Indian Ocean with a population of 1.3 million in 2023. Mauritius is a multiethnic nation with 68% South Asian, 27% African (Creole), 3% Chinese and 2% Franco Mauritians.^9^

Seven population-based cross-sectional surveys using standardised protocols were conducted since 1987 (1987, 1992, 1998, 2004, 2009, 2015 and 2021). The first three surveys were conducted in the same areas and 60% of the participants were examined in more than one survey, whereas the surveys thereafter were based on new random samples. In addition, in 2015, a random selection from the 1998 survey was resurveyed. We have previously published detailed descriptions of the survey methodology and data collection,^8 10^ and they are further described in online supplemental material. The participation rate has been over 85% in each survey.^8^

### Self-reported CVD

All participants were interviewed about their health history and living conditions. Ethnicity and current smoking were self-reported. Participants were asked about previous myocardial infarction, stroke and angina pectoris as told by a doctor. An affirmative answer to any of these questions was labelled as the presence of CVD. Since 2009, questions about previous coronary bypass surgery (CABG) and percutaneous coronary intervention (PCI) were included.

### Measurements and biochemistry

Anthropometric and blood pressure (BP) measurements were done by trained personnel. Body mass index (BMI) was calculated, and overweight and obesity were defined by ethnicity-specific cut-offs.^11^ Waist and hip circumferences were measured using standardised methods. See online supplemental material for details regarding BP measurements and devices.

Hypertension was defined as a systolic BP equal to or above 140 mm Hg and/or diastolic BP equal to or above 90 mm Hg and/or use of BP lowering drugs.

Blood sampling took place between 08.00 and 10.00 hours after an overnight fast. All participants not taking medication for diabetes or being pregnant had a 2-hour 75-gram oral glucose tolerance test. Fasting and 2-hour venous blood samples were centrifuged and separated immediately. Plasma glucose assays and adjustments have been described previously.^8 10^

The glucose tolerance status was determined according to the 2006 WHO criteria.^12^ See online supplemental material for details.

Total cholesterol was determined in fresh venous plasma by enzymatic methods locally in Mauritius. See Supplementary material for details regarding quality control and adjustments.

### ECG and coding

Participants aged 35–74 years underwent a 12-lead ECG recording in all surveys except in 2004 and in 2021. The capture was >98% of recordings being readable. All ECGs were coded according to the Minnesota Code classification in duplicate by a team led by one of the authors (AT).^13^ Discrepancies between the coders were solved by consensus. This classification was developed as a tool for comparison of ECGs in large clinical studies. The Minnesota Code is hierarchically based and represented by three numbers separated by dashes. The first number refers to the broad grouping (eg, Q waves=1 x-x) and the second and third numbers indicate severity. The Whitehall criteria were used for classifying ECG abnormalities into probable CHD (Minnesota Codes: 1–1 to 1–2 (large Q and QS)) and possible CHD (Minnesota Codes: 1–3 (small Q and QS), 4–1 to 4–4 (ST depression), 5-1 to 5–3 (T wave inversion and flat) and 7-1 (left bundle branch block)).^14^ Hence, probable CHD equals major ECG abnormalities and possible CHD equals minor ECG abnormalities.

### Ethics

All participants signed informed consent. The study protocols were reviewed and approved by the ethics committee of the Ministry of Health and Quality of Life, Mauritius, and by the Ethics at Baker IDI (previously International Diabetes Institute), Melbourne, Australia. The management of the data in Sweden was approved by the Swedish Ethical Review Authority (2023-07199-01).

### Statistical analysis

Most studied variables had very few missing data (table 1) and imputation was not done. Prevalence data were age-standardised by the direct method to the 2008 Mauritius population aged 35–75 years. Means and proportions are presented with 95% CIs. Associations of major demographic variables and major CVD risk factors with probable and possible CHD were analysed by univariable and multivariable logistic regression, reporting ORs with 95% CIs. To overcome potential differences between surveys and methods, survey-specific z-scores were calculated for fasting plasma glucose, 2-hour plasma glucose (2-hour glucose) and for total cholesterol.

**Table 1:** Baseline characteristics by year

Year	Missing (N)	1987	1992	1998	2004	2009	2015	2021
N		3395	5160	5013	3932	4836	4701	2940
ECG done (%)	96*	99	99	99	–	97	99	–
Age (years)	0	50.4(50.1 to 50.8)	50.1 (49.8 to 50.4)	51.1 (50.8 to 51.4)	51.0 (50.6 to 51.3)	51.9 (51.6 to 52.2)	55.8 (55.5 to 56.0)	54.3 (54.0 to 54.7)
Female sex (%)	0	53.4 (51.8 to 55.1)	53.9 (52.5 to 55.2)	55.7 (54.3 to 57.1)	60.6 (59.1 to 62.2)	54.0 (52.6 to 55.4)	55.7 (54.3 to 57.1)	54.1 (52.3 to 55.9)
Urban residency (%)	0	52.4 (50.7 to 54.1)	55.7 (54.3 to 57.1)	51.7 (50.4 to 53.1)	49.4 (47.8 to 50.9)	43.7 (42.3 to 45.1)	35.4 (34.0 to 36.7)	43.8 (42.0 to 45.6)
BMI (kg/m^2^)	238	24.0 (23.8 to 24.1)	25.3 (25.1 to 25.4)	25.5 (25.4 to 25.6)	25.9 (25.7 to 26.0)	25.8 (25.7 to 25.9)	26.3 (26.2 to 26.5)	26.7 (26.5 to 26.9)
Waist circumference (cm)	253	80.0 (79.7 to 80.4)	85.6 (85.3 to 85.8)	84.0 (83.7 to 84.3)	83.5 (83.2 to 83.8)	84.6 (84.3 to 84.9)	90.3 (90.0 to 90.6)	90.0 (89.6 to 90.4)
Systolic BP (mm Hg)	80	131 (130 to 132)	127 (126 to 127)	130 (130 to 131)	130 (129 to 130)	137 (136 to 138)	127 (127 to 128)	126 (126 to 127)
Diastolic BP (mm Hg)	85	80 (79 to 80)	77 (77 to 78)	76 (76 to 76)	80 (80 to 80)	81 (81 to 81)	80 (80 to 81)	79 (79 to 80)
Hypertension (%)	82	35.3 (33.7 to 36.9)	30.5 (29.3 to 31.8)	35.5 (34.2 to 36.8)	36.8 (35.3 to 38.3)	49.9 (48.5 to 51.3)	45.3 (43.9 to 46.8)	41.4 (39.6 to 43.2)
Smoking now (%)	0	32.2 (30.0 to 34.4)	28.4 (26.2 to 30.5)	22.0 (20.5 to 23.5)	36.2 (31.8–40.6)	24.3 (22.1 to 26.6)	16.6 (15.1 to 18.1)	24.3 (20.6 to 27.9)
Total serum cholesterol (mmol/L)	140	5.7 (5.6 to 5.7)	5.0 (5.0 to 5.1)	5.1 (5.1 to 5.1)	5.2 (5.1 to 5.2)	5.1 (5.1 to 5.2)	5.3 (5.3 to 5.3)	5.0 (4.9 to 5.0)
Fasting plasma glucose (mmol/L)	172	6.0 (6.0 to 6.1)	6.4 (6.3 to 6.5)	6.6 (6.5 to 6.6)	6.4 (6.3 to 6.5)	6.9 (6.8 to 7.0)	6.7 (6.6 to 6.8)	6.4 (6.4 to 6.5)
2-hour plasma glucose (mmol/L)	5552	7.7 (7.5 to 7.8)	7.8 (7.7 to 7.9)	7.9 (7.8 to 8.0)	6.6 (6.5 to 6.6)	7.8 (7.7–7.9)	7.7 (7.6 to 7.8)	6.6 (6.5 to 6.7)
*Glucose intolerance (%*)	526							
Diabetes		19.0 (17.7 to 20.3)	22.6 (21.5 to 23.8)	26.4 (25.2 to 27.7)	24.7 (23.4 to 26.1)	32.2 (30.9–33.5)	37.2 (35.8 to 38.6)	30.6 (28.9 to 32.3)
Pre-diabetes		29.0 (27.3 to 30.7)	30.3 (28.8 to 31.7)	27.7 (26.2 to 29.1)	29.0 (27.3 to 30.7)	39.7 (38.0–41.4)	33.7 (32.0 to 35.5)	26.7 (24.7 to 28.6)
*Ethnicity (%*)	12							
South Asian		63.3	66.1	70.5	74.8	73.6	76.3	79.4
Creole (African)		27	26.8	26.7	18	22.7	19.9	14.5
Chinese		9.7	7.1	2.8	7.1	3.6	3.8	6.1
Other		–	–	–	–	0.2	0.1	–

Values shown are numbers, means and proportions with 95% CIs. Prediabetes=impaired fasting glucose and/or impaired glucose tolerance; hypertension=systolic BP ≥140 mm Hg and/or diastolic BP ≥90 mm Hg and/or use of BP lowering drugs.

* Not done in 2004 and in 2021.

BMI, body mass index; BP, blood pressure.

The final model included age, sex, ethnicity, hypertension, total cholesterol, glycaemic status, BMI, smoking and self-reported history of CVD. Missing categorical data were treated as a separate category and were not reported. The statistical software SPSS V.28 was used (IBM Corp).

### Patient and public involvement

It was not appropriate or possible to involve patients or the public in the design, conduct or reporting of our research. However, all results have been reported to the Ministry of Health and Wellness in Mauritius.

## Results

Baseline characteristics of 29 977 participants are presented in table 1. The ethnic composition was representative of the population of Mauritius. Between 1987 and 2021, the prevalence of hypertension and diabetes increased while the prevalence of active smoking decreased. The mean BMI increased, whereas plasma total cholesterol decreased between 1987 and 1992 and did not change after that. The mean systolic BP was higher in 2009; more people had very high pressure (systolic BP >200 mm Hg) compared with other surveys. The prevalence of diabetes peaked in 2015 whereas the prevalence of prediabetes (impaired fasting glucose (IFG) and/or impaired glucose tolerance (IGT)) peaked in 2009.

Compared with newcomers, the mean age was higher for participants resurveyed in 1998 and in 2015, but not in 1992, (online supplemental table 1). The proportion of women did not differ between resurveyed participants and newcomers.

The prevalence of probable CHD or major ECG abnormalities showed no change over time, being 1.6% (1.2–2.1%) in 1987 and 1.7% (1.4–2.1%) in 2015, whereas the prevalence of possible CHD or minor ECG abnormalities decreased significantly from 23.7% (22.3–25.1%) in 1987 to 17.3% (16.2–18.3%) in 2015 (table 2). Probable CHD was more common in men, and possible CHD was more common in women. The prevalence of probable CHD remained stable in both sexes, whereas the prevalence of possible CHD decreased between 1987 and 2015 in women. The prevalence of probable and possible CHD did not differ between resurveyed participants and newcomers (online supplemental table 1), except for the prevalence of possible CHD in 1992, which was lower in newcomers. The prevalence of probable and possible CHD increased with age in all surveys (online supplemental figures 1 and 2) and in both sexes (data not shown) but did not differ between rural and urban residency (online supplemental table 2).

**Table 2:** Prevalence of CHD (95% CI) by sex and year

	All	Prevalence% (95% CI)	Men	Prevalence% (95% CI)	Women	Prevalence% (95% CI)
	N		N		N	
Probable CHD (major ECG abnormalities)						
All	432/22 634	1.7 (1.5 to 1.9)	288/10 263	2.5 (2.2 to 2.8)	144/12 371	1.0 (0.9 to 1.2)
1987	58/3348	1.6 (1.2 to 2.1)	41/1563	2.5 (1.7 to 3.2)	17/1785	0.9 (0.4 to 1.3)
1992	63/5091	1.2 (0.9 to 1.5)	45/2346	1.9 (1.4 to 2.5)	18/2745	0.6 (0.3 to 0.9)
1998	117/4955	2.2 (1.8 to 2.7)	72/2195	3.0 (2.3 to 3.7)	45/2760	1.5 (1.1 to 2.0)
2009	92/4682	1.7 (1.4 to 2.1)	59/2143	2.5 (1.8 to 3.1)	33/2539	1.0 (0.6 to 1.4)
2015	102/4558	1.9 (1.5 to 2.3)	71/2016	2.8 (2.1 to 3.5)	31/2542	1.1 (0.7 to 1.5)
Possible CHD (minor ECG abnormalities)						
All	4655/22 634	19.4 (18.9 to 20.0)	1503/10 263	13.6 (12.9 to 14.2)	3152/12 371	24 (23.2 to 24.7)
1987	797/3348	23.7 (22.3 to 25.1)	227/1563	14.1 (12.4 to 15.9)	570/1785	31.9 (29.7 to 34)
1992	852/5091	16.8 (15.8 to 17.8)	260/2346	11.2 (9.9 to 12.5)	592/2745	21 (19.5 to 22.6)
1998	1079/4955	21.3 (20.1 to 22.4)	308/2195	13.6 (12.1 to 15.0)	771/2760	27 (25.3 to 28.6)
2009	971/4682	19.8 (18.7 to 21.0)	361/2143	15.9 (14.4 to 17.5)	610/2539	22.8 (21.2 to 24.5)
2015	956/4558	17.3 (16.2 to 18.3)	347/2016	13.3 (11.9 to 14.8)	609/2542	19.9 (18.4 to 21.5)

The prevalence is age and sex standardised using the 2008 Mauritian population as standard.

CHD, coronary heart disease.

The prevalence of probable CHD was higher in men than in women irrespective of ethnicity (table 3). The prevalence of possible CHD was higher in women than in men irrespective of ethnicity, and the prevalence was higher in Creole women compared with South Asian and Chinese women.

**Table 3:** Prevalence of CHD (95% CI) by sex and ethnicity

	South Asian	Prevalence % (95% CI)	Creole (African)	Prevalence % (95% CI)	Chinese	Prevalence % (95% CI)
	N		N		N	
Probable CHD (major ECG abnormalities)						
All	315/15 875	1.8 (1.6 to 2.0)	102/5583	1.6 (1.2 to 1.9)	15/1164	1.2 (0.6 to 1.8)
Men	215/7242	2.7 (2.3 to 3.0)	64/2429	2.2 (1.6 to 2.8)	9/589	1.7 (0.6 to 2.7)
Women	100/8633	1.1 (0.9 to 1.3)	38/3154	1.0 (0.6 to 1.3)	6/575	0.8 (0.1 to 1.5)
Possible CHD (minor ECG abnormalities)						
All	3124/15 875	18.8 (18.2 to 19.4)	1328/5583	22.3 (21.2 to 23.4)	201/1164	15.4 (13.3 to 17.4)
Men	1030/7242	13.3 (12.6 to 14.1)	395/2429	15.1 (13.7 to 16.6)	78/589	10.7 (8.2 to 13.2)
Women	2094/8633	23.0 (22.1 to 23.9)	933/3154	27.4 (25.8 to 29.0)	123/575	19.6 (16.3 to 22.8)

The prevalence is age and sex standardised using the 2008 Mauritian population as standard.

CHD, coronary heart disease.

The prevalence of both probable and possible CHD was higher among participants with diabetes compared with those with normoglycaemia (table 4). Participants with prediabetes had a higher prevalence of possible CHD than those with normoglycaemia, whereas no difference in the prevalence of probable CHD was observed. Participants with hypertension had more often probable and possible CHD compared with those without (2.3 (2.0–2.6) vs 1.2 (1.0–1.4) and 25.7 (24.8–26.6) vs 15.3 (14.7–15.9), respectively). Participants with self-reported CVD had more often probable and possible CHD compared with those without (10.8 (9.1–12.6) vs 1.2 (1.1–1.4) and 35.6 (32.9–38.3) vs 18.6 (18.0–19.1), respectively).

**Table 4:** Prevalence of CHD (95% CI) by comorbidities

	Diabetes	Prevalence % (95% CI)	Prediabetes	Prevalence % (95% CI)	Normoglycaemia	Prevalence % (95% CI)	Hypertension	Prevalence % (95% CI)	History of CVD	Prevalence % (95% CI)
	N		N		N		N		N	
Probable CHD (major ECG abnormalities)										
All	205/6179	2.5 (2.1 to2.9)	87/5164	1.5 (1.2 to 1.8)	134/10 979	1.3 (1.1 to 1.5)	276/8881	2.3 (2.0 to 2.6)	150/1214	10.8 (9.1 to 12.6)
Men	139/2876	3.5 (2.8 to4.2)	61/2257	2.4 (1.8 to 3.0)	85/4984	1.8 (1.4 to 2.1)	191/4118	3.4 (2.9 to 4.0)	125/660	16.9 (14.0 to 19.7)
Women	66/3303	1.6 (1.2 to2.0)	26/2907	0.7 (0.4 to 1.0)	49/5995	0.8 (0.6 to 1.0)	85/4763	1.3 (1.0 to 1.6)	25/554	3.8 (2.2 to 5.4)
Possible CHD (minor ECG abnormalities)										
All	1521/6179	20.7 (19.7 to 21.7)	1166/5164	21.4 (20.2 to 22.5)	1898/10 979	17.8 (17.0 to 18.5)	2619/8881	25.7 (24.8 to 26.6)	524/1214	35.6 (32.9 to 38.3)
Men	576/2876	16.5 (15.2 to 17.9)	372/2257	15.5 (14.0 to 16.9)	528/4984	10.9 (10.1 to 11.8)	977/4118	20.7 (19.5 to 22.0)	254/660	28.9 (25.5 to 32.4)
Women	945/3303	24.2 (22.7 to 25.6)	794/2907	25.6 (24.0 to 27.2)	1370/5995	23.0 (22.0 to 24.1)	1649/4763	30.2 (28.9 to 31.5)	270/554	42.2 (38.1 to 46.3)

The prevalence is age and sex standardised using the 2008 Mauritian population as standard. Pre-diabetes includes both impaired fasting glucose (IFG) and/or impaired glucose tolerance (IGT).

CHD, coronary heart disease; CVD, cardiovascular disease.

The prevalence of self-reported history of CVD was higher in men than women, and the prevalence estimates did not change between 1987 and 2021 (online supplemental table 3). Of self-reported CVD, 80% were heart-related CVD, 12% were strokes and 8% were both heart-related and strokes. The proportion of stroke only decreased from 34% in 1987 to 6% in 2021.

After adjustments, age remained strongly associated with the presence of both probable and possible CHD (table 5). Women had a lower risk of probable CHD and a higher risk of possible CHD than men. Ethnicity was not associated with probable CHD after adjustments, whereas Creole ethnicity and female sex were significantly associated with the presence of possible CHD. Taking confounders into account, survey year did not associate with the prevalence of probable CHD, whereas survey year associated with reduced prevalence of possible CHD. In addition, hypertension, diabetes, IGT, current smoking and a self-reported history of CVD were independently associated with the presence of probable CHD, and hypertension, IGT, high total cholesterol and self-reported history of CVD were independently associated with the presence of possible CHD. In separate models where fasting and 2-hour glucose replaced glucose intolerance categories, fasting glucose was not independently associated with the presence of either probable or possible CHD (OR (95% CI) (0.81 (0.63 to 1.04) and 0.98 (0.91 to 1.07), respectively), whereas 2-hour glucose was significantly associated with both probable (1.30 (1.11–1.52)) and possible CHD (1.07 (1.01–1.13)).

**Table 5:** Association of risk factors with the presence of ECG changes indicating probable and possible coronary heart disease

		Probable CHD (major ECG abnormalities)			Possible CHD (minor ECG abnormalities)		
		N	Univariable	Multivariable	N	Univariable	Multivariable
Age, 10 year	35–44	46/7134	1.00		918/7134	1.00	1.00
	45–55	95/6749	**2.20 (1.55 to 3.13**)	**1.71 (1.19 to 2.46**)	1312/6749	**1.63 (1.49 to 1.79**)	**1.42 (1.29 to 1.56**)
	55–64	161/5619	**4.55 (3.27 to 6.32**)	**2.81 (1.98 to 4.01**)	1554/5619	**2.59 (2.36 to 2.83**)	**1.98 (1.79 to 2.18**)
	65–74	130/3132	**6.67 (4.75 to 9.37**)	**3.63 (2.50 to 5.27**)	1126/3132	**3.80 (3.44 to 4.20**)	**2.56 (2.29 to 2.87**)
Sex (women vs men)		432/22 634	**0.41 (0.33 to 0.50**)	**0.46 (0.36 to 0.58**)	4910/22 634	**1.81 (1.70 to 1.94**)	**1.88 (1.74 to 2.04**)
Survey year	1987	58/3348	1.00	1.00	832/3348	1.00	1.00
	1992	63/5091	0.71 (0.50 to 1.02)	0.73 (0.51 to 1.06)	890/5091	**0.64 (0.58 to 0.71)**	**0.61 (0.55 to 0.69**)
	1998	117/4955	1.37 (1.00 to 1.89)	1.20 (0.86 to 1.68)	1148/4955	0.91 (0.82 to 1.01)	**0.81 (0.72 to 0.90**)
	2009	92/4682	1.14 (0.82 to 1.58)	0.90 (0.64 to 1.28)	1035/4682	**0.86 (0.77 to 0.95**)	**0.69 (0.62 to 0.77**)
	2015	102/4558	1.30 (0.94 to 1.80)	0.85 (0.60 to 1.20)	1005/4558	**0.86 (0.77 to 0.95**)	**0.60 (0.54 to 0.67**)
Ethnicity	South Asian	315/15 867	1.00	1.00	3306/15 867	1.00	1.0
	Creole	102/5590	0.92 (0.73 to 1.15)	0.90 (0.71 to 1.14)	1395/5590	**1.26 (1.18 to 1.36**)	**1.18 (1.09 to 1.27**)
	Chinese	15/1165	0.64 (0.38 to 1.09)	0.70 (0.41 to 1.19)	207/1165	**0.82 (0.70 to 0.96**)	**0.79 (0.67 to 0.93**)
Hypertension	No	155/13 721	1.00	1.00	2109/13 721	1.00	1.00
	Yes	276/8881	**2.81 (2.30 to 3.42**)	**1.47 (1.18 to 1.84**)	2792/8881	**2.53 (2.37 to 2.69**)	**1.83 (1.7 to 1.97**)
Cholesterol (z-score)		431/22 584	1.05 (0.96 to 1.16)	1.06 (0.96 to 1.16)	4896/22 584	**1.14 (1.10 to 1.17**)	**1.06 (1.03 to 1.10**)
Fasting glucose (z-score)		430/22 486	**1.19 (1.11 to 1.28**)		4885/22 486	**1.13 (1.10 to 1.16**)	
2-hour glucose (z-score)		301/18 936	**1.26 (1.16 to 1.36**)		3938/18 936	**1.18 (1.14 to 1.21**)	
Glucose tolerance	NGT	134/10 979	1.00	1.00	1980/10 979	1.00	1.00
	IFG	15/1408	0.87 (0.51 to 1.49)	0.64 (0.37 to 1.10)	288/1408	**1.17 (1.02 to 1.34**)	1.05 (0.91 to 1.22)
	IGT	72/3756	**1.58 (1.19 to 2.11**)	**1.39 (1.03 to 1.88**)	932/3756	**1.50 (1.37 to 1.64**)	**1.14 (1.04 to 1.25**)
	DM	205/6172	**2.78 (2.23 to 3.47**)	**1.60 (1.26 to 2.04**)	1636/6172	**1.64 (1.52 to 1.77**)	**1.02 (0.94 to 1.11**)
BMI, kg/m^2^	<18.5	26/1073	1.00	1.00	207/1073	1.00	1.00
	18.5–22.9/18.5–24.9	130/6892	0.77 (0.51 to 1.19)	0.72 (0.46 to 1.13)	1255/6892	**0.93 (0.79 to 1.10**)	0.87 (0.74 to 1.04)
	23–27.4/25-29.9	175/8894	0.81 (0.53 to 1.23)	0.71 (0.45 to 1.11)	1954/8894	**1.18 (1.00 to 1.38**)	1.06 (0.90 to 1.26)
	27.5-/30-	100/5635	0.73 (0.47 to 1.13)	0.65 (0.41 to 1.05)	1458/5635	**1.46 (1.24 to 1.72**)	1.13 (0.95 to 1.35)
Smoking	Never/past smoking	304/17 835	1.00	1.00	4110/17 835	1.00	1.00
	Current smoking	126/4731	**1.58 (1.28 to 1.95**)	**1.32 (1.03 to 1.69**)	778/4731	**0.66 (0.60 to 0.72**)	1.02 (0.92 to 1.14)
Self-reported CVD	No	279/21 346	1.00	1.00	4272/21 346	1.00	1.00
	Yes	150/1214	**10.65 (8.65 to 13.10**)	**6.47 (5.17 to 8.10**)	629/1214	**4.30 (3.82 to 4.83**)	**3.38 (2.98 to 3.84**)

Numbers and ORs with 95% CIs (in parentheses) for variables associated with possible and probable CHD are shown. The multivariable model included age, sex, survey year, ethnicity, total cholesterol, smoking, BMI, hypertension, glucose status and history of CVD. Missing values for categorical variables were treated as a separate category to maintain the size of the dataset but were not reported. Z-score=1 SD increase calculated specific for each survey and sex. Glucose tolerance status was defined according to WHO guidelines and hypertension as systolic BP ≥140 mm Hg and/or diastolic BP ≥90 mm Hg and/or taking antihypertensive drugs. Ethnicity-specific cut-offs for BMI were used.

BMI, body mass index; BP, blood pressure; CHD, coronary heart disease; CVD, cardiovascular disease; DM, diabetes mellitus; IFG, impaired fasting glucose; IGT, impaired glucose tolerance; NGT, normal glucose tolerance.

## Discussion

We have shown that the prevalence of ECG abnormalities indicative of probable CHD remained unchanged over 28 years in the Mauritian population, a finding supported by a stable prevalence of self-reported history of CVD. However, the prevalence of possible CHD decreased in women. At the same time, significant increases in some of the known CVD risk factors were seen while some decreased. To our knowledge, this is the first repeated cross-sectional study monitoring the prevalence of CHD based on ECG readings over such a long period. Most studies reporting CHD based on ECG readings have been single cross-sectional studies performed in the ‘80s and ‘90s.^15^ We have previously reported the prevalence of CHD in a subset of participants from the 1987 survey.^16^

We hypothesised that the prevalence of CHD as indicated by ECG abnormalities may increase in parallel with the increasing prevalence of diabetes and hypertension in Mauritius. This was, however, not proven.

### ECG abnormalities and ethnicity and sex

Ethnic differences in susceptibility to develop atherosclerosis have been reported from the US multiethnic study of atherosclerosis (MESA).^17^ In our study, the prevalence of probable CHD was similar in each ethnic group, but possible CHD was more common among Creoles than other ethnicities, in accordance with previous studies.^18^

Population-based cross-sectional studies in India and in Pakistan have reported an increasing prevalence of CHD in both urban and rural populations.^19 20^ Q-waves indicating major ECG abnormalities were twice as common in men than in women, whereas minor ECG abnormalities were twice as common in women than in men.^20^ In Finland, the prevalence of ECG abnormalities indicating possible CHD has decreased during the last decades,^21^ whereas it has increased in the elderly. Of black South Africans with stroke, 16.7% had ECG abnormalities indicative of CHD,^22^ whereas the prevalence based on history of myocardial infarction and Q-waves differed substantially between black and white South Africans with diabetes, 4% versus 23%, respectively. Some of these differences could be attributed to differences in coding and combinations of codes used.

In this study, the urban and rural labelling was done by officials at the Ministry of Health without knowledge about the study results. Mauritius is densely populated, and only modest differences between urban and rural areas exist, and we did not find any urban-rural difference in the prevalence of CHD.

### ECG abnormalities and CHD

In the MESA study, participants without detectable coronary artery calcium and with minor ECG abnormalities did not have an increased risk of CHD.^23^ However, major ECG abnormalities were associated with a 3-fold increased risk of CHD events. ECG abnormalities improved the CHD risk prediction when added to risk factors. Others have shown that both minor and major ECG abnormalities were associated with an increased risk of CHD events in elderly people, with similar findings in white and black participants.^24^ Major and minor ECG abnormalities independently predicted CHD events in postmenopausal women.^25^

The decrease in the prevalence of possible CHD in women should be cautiously interpreted as the prevalence of possible CHD fluctuates more than the prevalence of probable CHD, reflecting the more transient nature of ST-T abnormalities compared with Q-waves. In healthy people, sex differences in ECG mainly affect QRS and T-wave amplitudes and duration in precordial leads.^26^ A drift in coding of ST-T abnormalities between 1987 and onwards in this study cannot be fully excluded, although the methodology and coders have remained unchanged during the entire period.

### Traditional CVD risk factors and CHD

The prevalence of traditional CVD risk factors is increasing in many LMICs. However, the impact of these risk factors on the development of CHD may differ among countries and ethnicities. Cholesterol is a major determinant for CVD, and more than 50% of the incidence of acute myocardial infarction (MI) is attributed to high cholesterol levels together with hypertension, smoking, obesity and diabetes.^27^ Notably, cholesterol levels decreased in Mauritius between 1987 and 1992, most probably due to a change of cooking oil.^28^ This decrease could possibly have stabilised the incidence of acute MI. In our study, traditional CVD risk factors were associated with both probable and possible CHD. Interestingly, 2-hour glucose, but not fasting glucose, was associated with probable and possible CHD. This is in line with previous reports showing that 2-hour glucose is associated with ECG abnormalities suggestive of CAD^29^ and is a better predictor for CVD morbidity and mortality than fasting glucose levels and HbA1c.^30^ In 2007, vital status was determined for all participants in the first three surveys (1987–1998), and 91% were traced. Altogether, 16% had died, and glucose intolerance (diabetes and IGT) was associated significantly with CVD mortality.^31^ Two-thirds of patients with an acute MI have glucose intolerance (newly diagnosed diabetes or IGT),^32^ but diabetes attenuates the male excess in risk of CVD and premature mortality.^33^

Men had a higher prevalence of both CHD according to ECG and CVD according to self-reported data. Myocardial infarctions occur earlier in men, and men have more often coronary atherosclerosis.^34^ The prevalence of CVD at each period is thus the net effect of risk factor burden, treatment effects and survival with the disease.

### The clinical phenotype of myocardial ischaemia

The presentation of the acute coronary syndrome (ACS) has changed over time, possibly due to changing risk profiles, from predominantly ST-segment elevation MI (STEMI) to non-ST-segment elevation MI (NSTEMI).^35^ The true prevalence of CHD in Mauritius may thus be higher than the observed cross-sectional data indicate. In addition, a substantial proportion of those with an acute MI die before reaching hospital, and the prevalence of Q-waves will thus underestimate the true occurrence of probable CHD in the population. Furthermore, the introduction of thrombolysis in the ‘90s may have led to a lesser development of Q-waves. However, we do not have any information about the use of thrombolysis in our cohort. Recently, an around-the-clock PCI service for acute STEMI has been set up in Mauritius, and hopefully, the prevalence of ischaemic myocardial damage (Q-waves) will decrease in the future. The period studied in this report does not include this major development in Mauritius.

Changes in the incidence of ACS and its presentation are associated with risk factor profiles, and we have shown that traditional cardiovascular risk factors either improved (smoking and cholesterol) or worsened (diabetes and hypertension) during the period studied. The net result could be a stable prevalence of CAD during the period studied.

In this study, the findings from ECG and self-reported data on history of CVD are congruent. Information about previous CVD was obtained by interview and not confirmed by clinical evidence. Therefore, self-reported data should be cautiously interpreted. MI, stroke, CABG and PCI are obvious events, whereas the presence of angina is subjective. The high prevalence of probable and possible CHD in people with self-reported history of CVD indicates that the self-reports were often true.

### Strengths and limitations

The strength of this large study in the multiethnic population of Mauritius is its size and a long observation period. We are not aware of any other similar population-based ECG data, collected and coded using standardised methodology over such an extended period. It is important to emphasise that all ECGs have been analysed and coded manually by the same coders. Some survey participants have attended survey examinations more than once, mainly in 1992, 1998 and in 2015, but they still represented the target population in their age group. We applied a uniform age-standardisation and thus bias by age was excluded. The surveys in 1987, 2009, 2015 (new) and 2021 were all based on independent population samples.

The prevalence estimates of probable CHD are probably underestimated due to survival bias, as the risk for dying in the acute phase of an MI was high in the prereperfusion era and ECG abnormalities were not verified via hospital files. Despite this limitation, the prevalence estimates of ECG abnormalities associated with major cardiovascular risk factors and with self-reported CVD indicate that ECG abnormalities could be used as a tool for monitoring changes in the prevalence of CVD in the population.

The unique population mix in Mauritius with three distinct ethnicities improves generalisability. However, and in contrast to our findings, the prevalence of MI is decreasing in many parts of the world together with a changing phenotype of MI, which probably reflects dynamic changes in the cardiovascular risk factor burden.^36^

## Conclusion

ECG abnormalities indicative of probable CHD have not increased in middle-aged people in the multiethnic population of Mauritius during the past 28 years, despite an increasing prevalence of hypertension and diabetes. ECG abnormalities indicative of possible CHD have decreased in women but not in men. Traditional risk factors were associated with ECG abnormalities indicating CHD. It is important to include ECG recordings in future surveys to properly detect the time lag between risk factor exposure and CHD. Furthermore, in keeping with the ECG findings, the prevalence of self-reported history of CVD has remained stable over 34 years. These findings emphasise the importance of monitoring the CVD risk factors over time. Since the prevalence of CHD has not decreased in Mauritius, prompt preventive measures should be further promoted.

## Supplementary material

10.1136/bmjopen-2024-087693 online supplemental file 1

## Data Availability

Data from the Mauritius NCD surveys are not freely available according to GDPR due to the presence of individual data. However, pseudonymised data can be shared after ethical evaluation and formal request (https://www.umu.se/en/brs/).
