# Supplementary material for: Trends in the prevalence of cardiovascular disease, defined as ECG abnormalities and/or self-reported events, in Mauritius between 1987 and 2021: analysis of data from seven large population-based surveys
Source: BMJ Open. 2025 Jun 16;15(6):e087693. doi: 10.1136/bmjopen-2024-087693 (PMC12314806; doi:10.1136/bmjopen-2024-087693)
Supplement: online supplemental file 1 [file bmjopen-15-6-s001.docx]

**Supplementary material**

**Trends in the prevalence of cardiovascular disease, defined as electrocardiogram abnormalities and/or self-reported events, in Mauritius between 1987 and 2021: analysis of data from seven large population-based surveys**

**Stefan Söderberg^1^, Hina Taki**^1^**, Sudhir Kowlessur^2^, Jonathan E. Shaw^2^, Dianna J Magliano^2^, Abdonas Tamosiunas^4^, George Alberti^5^, Paul Zimmet^6^, Jaakko Tuomilehto^7^**

^1^Department of Public Health and Clinical Medicine, Umeå University, Umeå, Sweden.

^2^Ministry of Health and Wellness, Port Louis, Mauritius.

^3^Baker Heart and Diabetes Institute, Melbourne, Australia.

^4^Institute of Cardiology, Lithuanian University of Health Sciences, Kaunas, Lithuania.

^5^Department of Endocrinology and Metabolism, St. Mary’s Hospital and Imperial College, London, UK.

^6^Department of Diabetes, Central Clinical School, Monash University, Melbourne, Australia.

^7^Population Health Unit, Finnish Institute for Health and Welfare, Helsinki, Finland; Department of Public Health, University of Helsinki, Helsinki, Finland; Diabetes Research Group, King Abdulaziz University, Jeddah, Saudi Arabia.

**Content**

1. Survey methodology.
2. Survey methods (diabetes classification, measurement of blood pressure and cholesterol)
3. References
4. Supplementary Table 1 (prevalence of CHD stratified by survey status (newcomer or repeated survey)
5. Supplementary Table 2 (prevalence of CHD stratified by residency (rural or urban)).
6. Supplementary Table 3 (prevalence of self-reported CVD by year and sex
7. Supplementary Figure 1
8. Supplementary Figure 2
9. **Survey methodology**

Mauritius is a subtropical island located in the South-western Indian Ocean with a population of approximately 1.3 million (2023). The multi-ethnic population consists of 68% individuals of South Asian origin (52% Hindu and 16% Muslim), 3% individuals of Chinese origin, 2% individuals with European origin, and 27% are of the general population, which mainly compromises of people with mixed African and Malagasy ancestry with some European and Indian admixture (Creoles).^1^

In 1987, 10 randomly selected (with probability proportional to size) population clusters and a purposely selected area of Chinatown in the capital, Port Louis, were surveyed (age range 25–74), and all eligible residents were invited to participate.^2^ In 1992 and 1998, the same clusters were resurveyed as well as an additional three clusters selected to assess if trends in disease and risk factor distribution observed in the study cohort also occurred in independent clusters (age range 25 years and older). In 1998, the Chinatown cluster was not surveyed. Altogether, 9,559 individuals participated in the first three surveys and 60% of participants took part in more than one survey.^3 4^

In 2004, a new study was performed, with similar methodology.

In 2009, nine index clusters were chosen with one randomly selected from each of the nine districts in Mauritius. Then, two neighbouring clusters were chosen surrounding the index cluster to form a “super cluster.” From this super cluster, one in three households was randomly selected and one adult per household was invited to participate.^5^ The same methodology was used in 2015 and in 2021 with new randomly chosen clusters forming “super cluster” as in 2009. In addition, a random sample from the 1998 survey was invited for a resurvey in 2015.

Participation rate has been over 85% in each survey. No sampling weights were used.

1. **Survey methods**

In the first four surveys, BP was measured with a standard mercury sphygmomanometer, and since 2009, BP was measured using an automated blood pressure monitor (Omron Digital Auto Blood Pressure Monitor SEM-1 in 2009 and Omron M7 in 2015). Hypertension was defined as a systolic BP equal to or above 140 mmHg and/or diastolic BP equal to or above 90 mmHg, and/or use of BP lowering drugs.

The glucose tolerance status was determined according to the 2006 World Health Organization criteria.^6^ Diabetes was classified on the basis of fasting plasma glucose ≥7.0 mmol/L or two-hour plasma glucose (2h glucose) ≥ 11.1 mmol/L or current treatment with insulin or oral glucose lowering drugs. Participants with a fasting glucose < 7.0 mmol/L and a 2h glucose ≥ 7.8 mmol/L to < 11.1 mmol/L were defined as having impaired glucose tolerance (IGT). Participants with a fasting glucose ≥ 6.1 mmol/L to < 7.0 mmol/L and a 2h glucose < 7.8 mmol/L were categorized as having impaired fasting glucose (IFG). Prediabetes was defined as IFG and/or IGT.

Total cholesterol was determined in fresh venous plasma by enzymatic methods locally in Mauritius. In the first three surveys, external quality assurance on every 10^th^ sample was undertaken in Newcastle-upon-Tyne, UK; cholesterol levels were consistently overestimated and were adjusted downwards using a regression equation. In 2015, a random sample of subjects (n=110) were tested for total cholesterol at Umeå University Hospital, Sweden; the correlation with values measured in Mauritius was 0.99.

1. **References**

1 Central Intelligence Agency (CIA), Washington DC, USA. The world factbook. [*https://wwwciagov/the-world-factbook/countries/*](https://wwwciagov/the-world-factbook/countries/) 2024.

2 Dowse GK, Gareeboo H, Zimmet PZ*, et al.* High prevalence of NIDDM and impaired glucose tolerance in Indian, Creole, and Chinese Mauritians. Mauritius Noncommunicable Disease Study Group. *Diabetes* 1990;39:390-6.

3 Söderberg S, Zimmet P, Tuomilehto J*, et al.* Increasing prevalence of Type 2 diabetes mellitus in all ethnic groups in Mauritius. *Diabet Med* 2005;22:61-8.

4 Söderberg S, Zimmet P, Tuomilehto J*, et al.* High incidence of type 2 diabetes and increasing conversion rates from impaired fasting glucose and impaired glucose tolerance to diabetes in Mauritius. *J Intern Med* 2004;256:37-47.

5 Magliano DJ, Söderberg S, Zimmet PZ*, et al.* Explaining the increase of diabetes prevalence and plasma glucose in Mauritius. *Diabetes Care* 2012;35:87-91.

6 WHO. Definition and diagnosis of diabetes mellitus and intermediate hyperglycaemia. *World Health Organization Geneva, Switzerland, Department of Non-communicable Disease Surveillance* 2006.

| 1. **Supplementary table 1. Prevalence of CHD stratified for survey status.** | | | | | | | | | | | |  |
| --- | --- | --- | --- | --- | --- | --- | --- | --- | --- | --- | --- | --- |
|  | | | | Probable CHD |  | |  | | Possible CHD | |  | |
|  | Age (years) | Female sex (%) |  | N | Prevalence (%) | |  | | N | | Prevalence (%) | |
|  |  |  |  |  |  |  | |  | |  | |  |
| ***Newcomers*** | |  |  |  |  |  | |  | |  | |  |
| 1992 | 49.8 (49.4–50.2) | 53.6 (51.4–55.7) |  | 30/2027 | 1.6 (1.0–2.1) |  | | 247/2027 | | 12.3 (10.8–13.7) | |  |
| 1998 | 47.4 (46.7–48.2) | 55.9 (52.4–59.4) |  | 14/776 | 1.7 (0.8–2.6) |  | | 164/776 | | 23.0 (20.0–25.9) | |  |
| 2015 | 53.9 (53.6–54.3) | 54.8 (53.0–56.6) |  | 56/2827 | 1.7 (1.3–2.2) |  | | 566/2827 | | 17.3 (15.9–18.7) | |  |
| All | 51.5 (51.3–51.8) | 54.5 (53.2–55.8) |  | 100/5630 | 1.7 (1.3–2.0) |  | | 977/5630 | | 16.4 (15.5–17.4) | |  |
|  |  |  |  |  |  |  | |  | |  | |  |
| ***Resurveyed*** | |  |  |  |  |  | |  | |  | |  |
| 1992 | 50.3 (49.9–50.7) | 54.1 (52.3–55.8) |  | 33/3064 | 1.0 (0.7–1.4) |  | | 605/3064 | | 19.7 (18.3–21.1) | |  |
| 1998 | 51.8 (51.4–52.1) | 55.6 (54.1–57.1) |  | 103/4179 | 2.2 (1.8–2.7) |  | | 915/4179 | | 20.9 (19.7–22.2) | |  |
| 2015 | 58.6 (58.2–59.0) | 57.0 (54.8–59.3) |  | 46/1731 | 2.1 (1.5–2.8) |  | | 390/1731 | | 17.6 (15.8–19.4) | |  |
| All | 52.6 (52.4–52.9) | 55.4 (54.4–56.4) |  | 182/8974 | 1.7 (1.5–2.0) |  | | 1910/8974 | | 19.6 (18.8–20.5) | |  |
|  |  |  |  |  |  |  | |  | |  | |  |

Supplementary table 1 shows mean age and proportion of female sex with 95% confidence intervals (in parentheses), numbers, and prevalence (%) of probable and possible CHD with 95% confidence intervals (in parentheses) based on ECG changes, stratified for survey status (newcomer or resurveyed). The prevalence is age and sex standardised using the 2008 Mauritian population as standard. CHD=coronary heart disease.

1. **Supplementary table 2. Prevalence of CHD stratified for residence.**

|  | Rural |  |  | Urban |  |
| --- | --- | --- | --- | --- | --- |
|  |  |  |  |  |  |
|  | N | Prevalence (%) |  | N | Prevalence (%) |
|  |  |  |  |  |  |
| ***Probable CHD (major ECG abnormalities)*** | | |  |  |  |
| All | 209/11 708 | 1.7 (1.4–1.9) |  | 223/10 822 | 1.7 (1.5–2.0) |
| Men | 146/5355 | 2.5 (2.1–2.9) |  | 142/4856 | 2.4 (2.0–2.8) |
| Women | 63/6353 | 0.9 (0.7–1.2) |  | 81/5966 | 1.2 (0.9–1.4) |
|  |  |  |  |  |  |
| ***Possible CHD (minor ECG abnormalities)*** | | |  |  |  |
| All | 238/11 708 | 19.6 (18.9–20.4) |  | 225/10 822 | 19.3 (18.6–20.1) |
| Men | 796/5355 | 14.1 (13.2–15.0) |  | 704/4856 | 13.2 (12.2–14.1) |
| Women | 1592/6353 | 23.9 (22.9–25.0) |  | 1549/5966 | 24.0 (23.0–25.1) |
|  |  |  |  |  |  |

Supplementary table 2 shows numbers and prevalence (%) with 95% confidence intervals (in parentheses) of probable and possible CHD based on the ECG changes stratified for residence (urban or rural). The prevalence is age and sex standardised using the 2008 Mauritian population as standard. CHD=coronary heart disease, and ECG=electrocardiogram.

1. **Supplementary table 3. Prevalence of self-reported CVD (95% CI) by survey year and sex**

|  |  |  | |  |  |  | |  |  |  | |  |
| --- | --- | --- | --- | --- | --- | --- | --- | --- | --- | --- | --- | --- |
|  |  | All |  | |  | Men |  | |  | Women |  | |
| Survey year |  | N | | Prevalence  % (95% CI) |  | N | | Prevalence  % (95% CI) |  | N | | Prevalence  % (95% CI) |
|  |  |  | |  |  |  | |  |  |  | |  |
| All |  | 1614/29 874 | | 4.8 (4.5–5.0) |  | 868/13 344 | | 5.7 (5.3–6.1) |  | 746/16 529 | | 3.8 (3.6–4.1) |
| 1987 |  | 105/3389 | | 3.0 (2.4–3.5) |  | 65/1578 | | 3.9 (2.9–4.9) |  | 40/1811 | | 2.0 (1.4–2.7) |
| 1992 |  | 199/5150 | | 3.9 (3.4–4.4) |  | 109/2375 | | 4.7 (3.8–5.5) |  | 90/2775 | | 3.1 (2.5–3.8) |
| 1998 |  | 300/5010 | | 5.7 (5.0 –6.3) |  | 135/2220 | | 5.7 (4.7–6.7) |  | 165/2790 | | 5.4 (4.6–6.2) |
| 2004 |  | 234/3932 | | 5.6 (4.9–6.4) |  | 103/1556 | | 6.0 (4.8–7.2) |  | 131/2375 | | 5.1 (4.3–6.0) |
| 2009 |  | 263/4767 | | 4.9 (4.3–5.5) |  | 154/2191 | | 6.2 (5.2–7.2) |  | 109/2576 | | 3.5 (2.8–4.2) |
| 2015 |  | 373/4691 | | 5.8 (5.1–6.4) |  | 209/2078 | | 7.2 (6.1–8.3) |  | 164/2613 | | 4.4 (3.6–5.2) |
| 2021 |  | 140/2935 | | 3.7 (3.1–4.4) |  | 93/1346 | | 4.9 (3.7–6.0) |  | 47/1589 | | 2.3 (1.6–3.0) |

Supplementary table 3 shows numbers and prevalence (%) with 95% confidence intervals (CI) in parentheses.

CVD is defined as a history of angina, myocardial infarction or stroke **as told by a physician**. Previous coronary by-pass (CABG) or percutaneous coronary intervention (PCI) were included from the 2015 survey. The prevalence is age and sex standardised using the 2008 Mauritian population as standard.

1. **Supplementary Figure 1 – Probable CHD by age-groups**

Prevalence (%) of probable CHD (major ECG abnormalities) stratified for age-groups (10-year) and survey year.

1. **Supplementary Figure 2 – Possible CHD by age-groups**

Prevalence (%) of possible CHD (minor ECG abnormalities) stratified for age-groups (10-year) and survey year.
