# Supplementary material for: Trends in the prevalence of cardiovascular disease, defined as ECG abnormalities and/or self-reported events, in Mauritius between 1987 and 2021: analysis of data from seven large population-based surveys
Source: BMJ Open. 2025 Jun 16;15(6):e087693. doi: 10.1136/bmjopen-2024-087693 (PMC12314806; doi:10.1136/bmjopen-2024-087693)
Supplement: Supplementary file 2 [file bmjopen-2024-087693.reviewer_comments.pdf]

## PEER REVIEW HISTORY

BMJ Open publishes all reviews undertaken for accepted manuscripts. Reviewers are asked to complete a checklist review form and are provided with free text boxes to elaborate on their assessment. These free text comments are reproduced below.

### ARTICLE DETAILS

**Title (Provisional)**

Trends in the prevalence of cardiovascular disease, defined as electrocardiogram abnormalities and/or self-reported events, in Mauritius between 1987 and 2021: analysis of data from seven large population-based surveys

**Authors**

Söderberg, Stefan; Taki, Hina; Kowlessur, Sudhir; Shaw, Jonathan E; Magliano, Dianna; Tamosiunas, Abdonas; Alberti, George; Zimmet, Paul.; Tuomilehto, Jaakko

---

### VERSION 1 - REVIEW

---

| | |
|---|---|
| **Reviewer** | **1** |
| **Name** | **Kulkarni, Aparna** |
| **Affiliation** | **Steven and Alexandra Cohen Children's Medical Center, Pediatrics** |
| **Date** | **12-Dec-2024** |
| **COI** | **None** |

---

This is a well written paper and an interesting population health analysis. My suggestion is to accept with minor revisions. I have the following further comments for improvement:

• The authors report that missing data was omitted. This may result in bias and under reporting of the true prevalence, if persons with true CHD or persons who died of CHD did not report their data or participate in the survey. Non-ST segment MI could also be potentially missed.

• Was there a specific change implemented in Mauritius after 1992 that may have accounted for the no change in total cholesterol level of the populations studied. How could we eliminate participation bias?

• Could the authors speculate for the decrease in CHD prevalence in select populations, as noted.

• Suggest removing the last 2 sentences in discussion regarding generalising of the findings of this population in Mauritius to the world population. The global prevalence of CHD is in fact, increasing.

• Rare repetition of words/ grammatical errors was noted. Suggest thorough editing.

| | |
|---|---|
| **Reviewer** | **2** |
| **Name** | **Boonstra, Machteld J** |
| **Affiliation** | **Amsterdam UMC Locatie AMC, Cardiology** |
| **Date** | **16-Mar-2025** |
| **COI** | **None** |

I would like to congratulate the authors with their work, the work described in the article is an immense effort to obtain the measurements over a long period and to allow for insight in population based CVD-information in such an elaborate way. Through the insights obtained, the study is a great addition to the current base of literature. I however do have some questions regarding specifics of your study.

Self-reported outcomes can present several challenges in terms of reliability and validity. Individuals may not accurately remember past events, experiences, or symptoms, especially when asked to report over long periods of time or interpret the question differently based on personal understanding and background, which may lead to inconsistent answers. There is a lack of objectivity and differences in responses can be obtained depending on the level of understanding. Therefore, I was wondering if (a subset of) the self-reported outcomes were validated in your study? Was additional information from hospitals / general practitioners obtained to check both positive and negative cases of self-reported CHD?

There is a 2% dip seen in the 65-74 group comparing 1987 to 1992 in supplementary figure 1, then an increase is seen in the probable CHD and then it decreases again for both 55-64 and 65-74 age groups. Do the authors know what the difference could be for this first decrease could be whereafter it increases again? Was the survey done in a different way or can it be explained by another factor?

When sending out the request to participate in the survey, did the authors straify for birth year and thus the mean age of the participants only is affected by the response rate?

There seems to be quite some missingness in the 2-hour plasma glucose measurement, was this evenly spread over the years regarding the missingness or were there specific years which also dominated this number? How is this caused?

The question in the discussion: “Do these ECG changes predict the true presence of CHD?” makes it a bit unclear. Would suggest to rephrase that based on the found ECG changes, it remains unresolved that the ECG changes found predict the true presence of CHD. Additionally, the authors did include self-reported CHD, but will the database be also supplemented with hospital based outcomes or information from GPs?

The authors indicate “A drift in coding of ST-T changes between 1987 onwards in our study cannot be fully excluded although the methodology and coders have remained unchanged ruing the entire period”. What do the authors mean by this? In the time period, also experience may have contributed to a shift in ECG evaluation, right?

Besides the Minnesota review of the data, were also standard ECG-measurements such as QRS duration, Q-wave amplitudes, QRS-axis and QTC timing included to also allow for comparison to such measurements, besides the Minnesota codes?

What is defined as a major (possible) versus minor (probable) ECG change? Is that only the Whitehall criteria as stated in the methods? Why did the authors not take into account the occurrence of atrial fibrillation? As there may be a direct interaction between the occurrence of AF and CHD.

Additionally, I’m not entirely sure if the term ECG changes should be used in the current study. The study describes a population based screening, but not necessarily follow-up study and therefore using the term ECG changes, referring to a change from the a previous ECG to the current one is a bit misleading. I would suggest to rephrase to ECG observations.

The authors indicate that in 2015, a random subset of 1998 was resurveyed in the methods. What were the findings there? How did the demographics besides age, but including for example self-reported CHD and the other measurements like diabetes and overall ECGs change over time in this group? Additionally, the authors indicate that 60% of the participants were resurveyed, did the authors also look into ECG changes in individuals over time specifically in these participants, besides assigning to probable and possible CHD? Were there for example more conduction delays found or more AF?

Regarding the STROBE checklist, it is provided, but it seems not to have been completed nor referred to from the main manuscript. It needs to be indicated how each item applies to your study and detailed information on how you addressed each point in your study should be described. The checklist was included, but the authors do not have seem to take it into account.

Minor suggestions

- In table 1, it is indicated that 96 ECGs were missing, excluding the year of 2004 and 2021. Would it not be better to also indicate this the similar way for Pulse, as it seems to be the case that the missingness is dominated by the missing measurements of 1987?

- The sentence “Missing data was Prevalence data were age-standardized by the direct method to the 2008 Mauritius population aged 35-75 years.” Is not completely clear. Should “Missing data was” be removed here?

- I would suggest to include the information which is indicated in the results with “(data not shown)” in the supplementary figures or remove the statements from the results section.

- There is a typo in the sentence: "...CHD compared with those without (10.8 (9.1–12.6) vs. 1.2 (1.1–1.4), and 35.6 (32.9–38.3) vs. 18.6 (18.0–19.1, respectively).". It seems that the closing bracket is missing after 19.1. Page 8 manuscript.

- There are quite some abbreviations in the text, for readability I would suggest to remove IGT, FPG and 2-hPG.

- The discussion may benefit from using subsections.

---

## VERSION 1 - AUTHOR RESPONSE

Reviewer: 1
Dr. Aparna Kulkarni, Steven and Alexandra Cohen Children's Medical Center
Comments to the Author:
This is a well written paper and an interesting population health analysis. My suggestion is to accept with minor revisions. I have the following further comments for improvement:

We do appreciate the positive statement regarding the quality of our paper.

• The authors report that missing data was omitted. This may result in bias and under reporting of the true prevalence, if persons with true CHD or persons who died of CHD did not report their data or participate in the survey. Non-ST segment MI could also be potentially missed.

This comment contains several aspects.

1. Missingness, as can be seen from Table 1, the actual number of missingness is extremely low. **In the footnote to Table 4, it is stated that persons with missing data gets a separate category** in the categorical analysis, the reason is to use the full dataset, which will maintain the statistical power. Furthermore, the highest number of missing is for pulse which was not registered in all surveys and 2h Glucose (OGTT not done if high fasting glucose or known diabetes). Pulse was not used in any analysis and has been omitted from Table 1, and missing 2h glucose did not affect substantially the coding of diabetes (as other criteria were present).
2. Missingness in ECGs is extremely low as seen in Table 1, only 96 out of thousands.
3. We fully agree with the reviewer that the main reason for underestimation of the prevalence is survival bias; only those surviving their MI have the potential to attend the next survey, and furthermore, the willingness may be low due to already established contact with health care. **This is discussed on page 14**.
4. We are fully aware that minor MIs, like NSTEMI may not leave any scars (Q waves) or repolarisation changes (ST-T) and would be missed. However, the self-reported (doctors-told) prevalence of CVD was also stable. Furthermore, the strong association between self-reported CVD and the presence of ECG changes indicate that the numbers given are rather robust. **This is discussed on page 14**.

• Was there a specific change implemented in Mauritius after 1992 that may have accounted for the no change in total cholesterol level of the populations studied. How could we eliminate participation bias?

Thanks for this valuable comment, in fact a decrease in cholesterol levels in Mauritius has been described, and could have possibly stabilised the incidence of MI. This is discussed in page 13, and reference is given. Together with our recent publication from GCVRC showing the impact of major risk factors for incident MI.

As stated on page 7, the participation rate has been extremely high, more than 85%. The large sample sizes, the random samples, and the high participation rate should give robust estimates, but we fully acknowledge that there is always as risk for selection bias. In this case, mainly survival bias, which is discussed in the manuscript.

• Could the authors speculate for the decrease in CHD prevalence in select populations, as noted.

Referring to Table 2, which contain our main findings, no decrease (or increase) in the age-standardises prevalence of probable CHD. The decrease of possible CHD (minor abnormities) in women is more difficult to evaluate, and is already discussed on page 13

• Suggest removing the last 2 sentences in discussion regarding generalising of the findings of this population in Mauritius to the world population. The global prevalence of CHD is in fact, increasing.

With all due respect, we ask for the permission to keep these sentences. We do not say that the findings are applicable all over, BUT that the unique population mix in Mauritius with three distinct ethnicities improves the generalisability. Btw, the prevalence is decreasing in many countries (like my own, Sweden) and that the phenotype of acute MI has changed.

• Rare repetition of words/ grammatical errors was noted. Suggest thorough editing.

Done to the best of our knowledge, please correct if any left.

Reviewer: 2
Dr. Machteld J Boonstra, Amsterdam UMC Locatie AMC, UMC Utrecht
Comments to the Author:

I would like to congratulate the authors with their work, the work described in the article is an immense effort to obtain the measurements over a long period and to allow for insight in population based CVD-information in such an elaborate way. Through the insights obtained, the study is a great addition to the current base of literature. I however do have some questions regarding specifics of your study.

We do appreciate the positive statement regarding the quality of our paper.

Self-reported outcomes can present several challenges in terms of reliability and validity. Individuals may not accurately remember past events, experiences, or symptoms, especially when asked to report over long periods of time or interpret the question differently based on personal understanding and background, which may lead to inconsistent answers. There is a lack of objectivity and differences in responses can be obtained depending on the level of understanding. Therefore, I was wondering if (a subset of) the self-reported outcomes were validated in your study? Was additional information from hospitals / general practitioners obtained to check both positive and negative cases of self-reported CHD?

We fully agree with this insightful comment, and this is discussed on page 15. Notably, MI, stroke, PCI and CABG are more objective events whereas angina is subjective. Furthermore, all this information was obtained by interview (remembering that many (in early years) were illiterate. And that events should have been "told by a doctor" to be counted. No validation has been done vs hospital records (however, the first author has adjudicated causes of death stated on death certificates vs hospital files with good agreement, see reference 32).

There is a 2% dip seen in the 65-74 group comparing 1987 to 1992 in supplementary figure 1, then an increase is seen in the probable CHD and then it decreases again for both 55-64 and 65-74 age groups. Do the authors know what the difference could be for this first decrease could be whereafter it increases again? Was the survey done in a different way or can it be explained by another factor?

We acknowledge the rather shaky appearance of the age specific prevalence. However, this should be interpreted very cautiously, the play of chance, small numbers and non-age adjusted. The main message is that the prevalence increases with age, and that is the only comment given in the results, all other comment would be speculative.

When sending out the request to participate in the survey, did the authors stratify for birth year and thus the mean age of the participants only is affected by the response rate?

As stated in the supplementary material, participants were randomly chosen from the randomly chosen household, within the randomly chosen cluster. Thus, no participants were at priori chosen. Furthermore the 10-years age strata from 35 to 75 contained between ≈ 7300 subjects aged 35 to 45 and ≈ 3200 aged 65 to 75. We do believe that these large numbers will give an unbiased prevalence (which is also standardised vs the Mauritian population).

There seems to be quite some missingness in the 2-hour plasma glucose measurement, was this evenly spread over the years regarding the missingness or were there specific years which also dominated this number? How is this caused?

As stated to reviewer 1, this is very easily explained. Every participant was invited in the morning and an OGTT was offered everyone, but not done if the person has diabetes, is

pregnant and/or has a high fasting glucose level (newly detected diabetes). This missingness did not affect the coding of diabetes as other criteria were most often present. Of course, some persons could not stay for 2 hours, and dropped of before the 2h glucose was taken, but that was a minority.

The question in the discussion: “Do these ECG changes predict the true presence of CHD?” makes it a bit unclear. Would suggest to rephrase that based on the found ECG changes, it remains unresolved that the ECG changes found predict the true presence of CHD.

Thanks, has been removed and replaced with subheadings.

Additionally, the authors did include self-reported CHD, but will the database be also supplemented with hospital based outcomes or information from GPs?

For this study no but was done for the previous study on CVD mortality in subjects participating the first three surveys (reference 32).

The authors indicate “A drift in coding of ST-T changes between 1987 onwards in our study cannot be fully excluded although the methodology and coders have remained unchanged ruing the entire period”. What do the authors mean by this? In the time period, also experience may have contributed to a shift in ECG evaluation, right?

Until now, ECG interpretation has been mainly done manually. In this study, Professor Abdonas Tamosiunas from Kaunas, Lithuania, has done ALL codings, together with a colleague, and discrepancies have been solved by consensus. Prof Abdonas was responsible for all ECG coding in the MONICA study in the eighties and is an authority within the field. In my opinion, it can’t be better than that (tomorrow by AI, but that is beyond this study).

Professor Abdobas comment on this remarque; *“of course, coding of such large number of ECGs the coders gain larger experience, and it could slightly change coding results between different in time coding episodes”.*

Besides the Minnesota review of the data, were also standard ECG-measurements such as QRS duration, Q-wave amplitudes, QRS-axis and QTC timing included to also allow for comparison to such measurements, besides the Minnesota codes?

The amplitude of Q-waves is used by the coders for determining if the finding represent a probable or possible CHD. The other measurements are available for last survey, and comparisons over time is thus not possible and therefore omitted from this analysis.

What is defined as a major (possible) versus minor (probable) ECG change? Is that only the Whitehall criteria as stated in the methods?

Correct! Based on grouping of the ECG changes according to Whitehall criteria.

Why did the authors not take into account the occurrence of atrial fibrillation? As there may be a direct interaction between the occurrence of AF and CHD.

Only available in the surveys from 2009 and 2015 and omitted from this study as comparisons over time is not possible. Analysis will be done later. Furthermore, in this population with high prevalence of badly treated hypertension, we believe that AF may have many other explanations than CHD.

Additionally, I'm not entirely sure if the term ECG changes should be used in the current study. The study describes a population based screening, but not necessarily follow-up study and therefore using the term ECG changes, referring to a change from the a previous ECG to the current one is a bit misleading. I would suggest to rephrase to ECG observations.

We do agree, we have changed it to ECG abnormalities throughout the manuscript.

The authors indicate that in 2015, a random subset of 1998 was resurveyed in the methods. What were the findings there? How did the demographics besides age, but including for example self-reported CHD and the other measurements like diabetes and overall ECGs change over time in this group? Additionally, the authors indicate that 60% of the participants were resurveyed, did the authors also look into ECG changes in individuals over time specifically in these participants, besides assigning to probable and possible CHD? Were there for example more conduction delays found or more AF?

We have carefully studied the prevalence of ECG abnormalities in those seen for the first time (newcomers) and those resurveyed, see Supplementary table 1. No difference in the prevalence.

Regarding the STROBE checklist, it is provided, but it seems not to have been completed nor referred to from the main manuscript. It needs to be indicated how each item applies to your study and detailed information on how you addressed each point in your study should be described. The checklist was included, but the authors do not have seem to take it into account.

Thanks, and we admit that the comments were a bit short. In this version, we have tried to be more explicit.

Minor suggestions
- In table 1, it is indicated that 96 ECGs were missing, excluding the year of 2004 and 2021. Would it not be better to also indicate this the similar way for Pulse, as it seems to be the case that the missingness is dominated by the missing measurements of 1987?

Pulse has been deleted.

- The sentence "Missing data was Prevalence data were age-standardized by the direct method to the 2008 Mauritius population aged 35-75 years." Is not completely clear. Should "Missing data was" be removed here?

Typo, removed.

- I would suggest to include the information which is indicated in the results with "(data not shown)" in the supplementary figures or remove the statements from the results section.

One is deleted, one left as it increases readability.

- There is a typo in the sentence: "...CHD compared with those without (10.8 (9.1–12.6) vs. 1.2 (1.1–1.4), and 35.6 (32.9–38.3) vs. 18.6 (18.0–19.1, respectively).". It seems that the closing bracket is missing after 19.1. Page 8 manuscript.

Thanks, corrected.

- There are quite some abbreviations in the text, for readability I would suggest to remove IGT, FPG and 2-hPG.

We keep IGT as that is clearly defined in supplement and is an integral part of prediabetes. FPG and 2-hPG have been replaced with fasting glucose and 2h glucose, respectively.

- The discussion may benefit from using subsections.

Thanks, have been added, which hopefully increase readability. Related to that, some paragraphs have changed order to increase clarity.

---

## VERSION 2 - REVIEW

---

| | |
|---|---|
| **Reviewer** | **1** |
| **Name** | **Kulkarni, Aparna** |
| **Affiliation** | **Steven and Alexandra Cohen Children's Medical Center, Pediatrics** |
| **Date** | **04-May-2025** |
| **COI** | |

---

I thank the authors for addressing the comments satisfactorily in the revision.

A few minor additional comments –

• Title: Please amend the title to remove its declarative nature as recommended by the journal - Prevalence of cardiovascular disease in Mauritius between 1987 and 2021 from seven large population-based surveys of self-reported cardiovascular events and electrocardiogram abnormalities

• Suggest editing the last 2 sentences in discussion. Since the authors feel strongly about this statement, it is my suggestion to reframe the sentences as stated in their response as follows instead: “The unique population mix in Mauritius with three distinct ethnicities improves the generalisability. The prevalence is decreasing in many countries such as Sweden and the phenotype of acute MI is also changing worldwide.”

---

| | |
|---|---|
| **Reviewer** | **2** |
| **Name** | **Boonstra, Machteld J** |
| **Affiliation** | **Amsterdam UMC Locatie AMC, Cardiology** |
| **Date** | **03-May-2025** |
| **COI** | |

---

The authors have sufficiently improved the manuscript.

---

## VERSION 2 - AUTHOR RESPONSE

Reviewer 1 and 2,
We are moist thankful for the many good suggestions and improvements, see below.

• Title: Please amend the title to remove its declarative nature as recommended by the journal - Prevalence of cardiovascular disease in Mauritius between 1987 and 2021 from seven large population-based surveys of self-reported cardiovascular events and electrocardiogram abnormalities

Changed according to the Editors suggestion.

• Suggest editing the last 2 sentences in discussion. Since the authors feel strongly about this statement, it is my suggestion to reframe the sentences as stated in their response as follows instead: “The unique population mix in Mauritius with three distinct ethnicities improves the generalisability. The prevalence is decreasing in many countries such as Sweden and the phenotype of acute MI is also changing worldwide.”

Thanks, I agree that the pattern differs worldwide.
The text is changed accordingly, see below, plus a reference describing that the change in Sweden is mainly due to improved risk profile in the population.

The unique population mix in Mauritius with three distinct ethnicities improves generalisability.
However, and in contrast to our findings, the prevalence of MI is decreasing in many parts of the world together with a changing phenotype of MI, which probably reflects dynamic changes in the cardiovascular risk factor burden.[36]
